# Evaluation of sampling methods for effective detection of infected pig farms during a disease outbreak

**DOI:** 10.1371/journal.pone.0241177

**Published:** 2020-10-22

**Authors:** Yoshinori Murato, Yoko Hayama, Yumiko Shimizu, Kotaro Sawai, Takehisa Yamamoto

**Affiliations:** Viral Disease and Epidemiology Research Division, National Institute of Animal Health, National Agriculture and Food Research Organization, Tsukuba, Ibaraki, Japan; University of Nicolaus Copernicus in Torun, POLAND

## Abstract

Emergency surveillance following an outbreak of transboundary animal diseases such as classical swine fever (CSF), is conducted to find another new infection as early as possible. Although larger sample sizes can help achieve higher disease surveillance sensitivity, the sample size is limited by the availability of resources in an emergency situation. Moreover, the recent CSF outbreak reported in Japan was associated with fewer clinical signs; this emphasizes the importance of detecting infected farms by surveillance. In this study, we aimed to identify effective and labor-efficient sampling methods showing high probabilities of detecting infection, by simulating infection and sampling in pigsties. We found that impartial sampling, which involves selection of pigs to be sampled from the four corners and the center of the pigsty, and random sampling showed comparable probabilities of detection. Impartial sampling involves sample collection without pig identification and random selection. Owing to its simplicity, impartial sampling is labor-efficient and thus a possible substitute for random sampling. In a group-housing pigsty, testing five pigs from five pens showed a higher detection probability than testing five pigs from one pen. These results suggest preferable surveillance methods for conducting emergency surveillance of infectious diseases.

## Introduction

When an outbreak of transboundary animal disease occurs in a free country, containment measures to prevent further spread are employed in the infected farms and surrounding areas. In addition, surrounding and epidemiologically related farms are subjected to emergency surveillance to detect other infected farms and to ensure their freedom from infection. If infected animals fail to show characteristic clinical signs, such as formation of vesicles on the mouth in foot and mouth disease or rapid increase in mortality rates in highly pathogenic avian influenza, finding infected animals should be achieved by conducting surveillance via specimen sampling and laboratory tests.

Classical swine fever (CSF) is a viral disease of pigs and boars, that is characterized by high mortality rates and varied clinical signs such as high fever and cyanosis of the skin [1]. Since its infectivity is very high, farmers consider CSF to be one of the most important pig diseases, and freedom from this disease needs to be approved by the International Animal Health Organization (OIE) [2]. After employing national eradication program for decades, Japan had declared CSF eradicated in 2007, and it has been approved as a CSF free country by the OIE in 2015. However, for the first time since its last outbreak in 1992, CSF re-emerged in September 2018 [3]. After the detection of the first case, all animals in the infected farms were slaughtered, and movement of animals and animal products within a 3 km radius (movement control area) and shipment within a 3–10 km radius (shipment control area) were prohibited [3, 4]. In addition, clinical inspections and antibody and antigen tests are conducted in all farms inside the movement control area on days 0, 17, and 26 after the detection of the infected farm, to find additional infected farms [3, 4]. The CSF virus (CSFV) isolated from infected farms during the 2018 outbreak belonged to serotype 2.1, which had previously been identified in cases reported from Asian countries including China, India, and South Korea, South Africa, and European countries including Germany and Lithuania [1, 5–7]. This type of CSFV does not reportedly cause severe clinical signs such as skin hemorrhage, diarrhea, and death, but generally causes non-specific signs such as intermittent fever, depression, wasting, and diffuse dermatitis [1, 3, 8]. Therefore, serological surveillance of surrounding and epidemiologically related farms is essential to find more infected animals as early as possible.

A higher sensitivity of serological surveillance is assured by a higher prevalence rate and a larger sample size. Thus, the sample size that ensures the sensitivity of surveillance depends on the design prevalence and test sensitivity. Although the sample size is calculated assuming that the distribution of infected animals and that of the sampled animals are both homogenous [9, 10], these assumptions may not reflect the real setting. Since the infection spreads to adjacent animals from the source animal, random sampling must be conducted carefully to avoid missing the infected animals, especially in the emergency situation. In addition, the availability of resources to sampling personnel and testing laboratories limit the sample size. In the case of the CSF outbreak in Japan, the minimum sample size for antigen/antibody testing was defined as five pigs/pigsty to enable simultaneous testing in all farms surrounding multiple infected farm sites [4]. To achieve maximum sensitivity under realistic conditions by testing a limited number of samples in potentially infected farms, the proper method of sampling should be encouraged among relevant official veterinarians, who conduct the sampling.

This study aimed to explore effective sampling methods for emergency surveillance with higher probabilities of detecting infection using limited sample sizes, by simulating infection patterns and sampling in pigsties. The effects of the distribution of infected pigs in a pigsty and those of the pigsty structure on the probability of detection were also examined. In addition, considering the differences in sampling methods used for individual-stall housing (for sows) and group housing (for fattening pigs), these two types of pigsties were included in the simulation. The probability of detecting at least one infected pig when five samples were tested was compared between random sampling and alternative sampling methods. Our results will provide scientific evidence to help veterinarians promote the use of more sensitive sampling methods during emergency surveillance.

## Materials and methods

### Overview of the study

According to the Monte Carlo test method, infection and sampling were repeatedly simulated in a virtual pigsty under different scenarios, for conducting surveillance. The sensitivity of the surveillance method in detecting infection was calculated as a proportion of iterations, with one or more detection of infected pigs in each scenario. Two infection patterns, four sampling methods, and two pigsty types were included in the simulation scenarios (Table 1).

**Table 1:** List of scenarios evaluated by Monte Carlo simulation.

No.	Type of the model pigsty	Pattern of infection	Sampling method	Number of tested pig(s) per stall or pen	Number of tested stall(s) or pen(s)
**1**	Stall-housing pigsty	Scattered infection	Random sampling	1	5
**2**			Vertical line sampling	1	5
**3**		Localized infection	Random sampling	1	5
**4**			Vertical line sampling	1	5
**5**			Horizontal line sampling	1	5
**6**			Impartial sampling	1	5
**7**	Group-housing pigsty	Scattered infection	Random sampling	1	5
**8**			Vertical line sampling	1	5
**9**		Localized infection	Random sampling	1	5
**10**				5	1
**11**			Vertical line sampling	1	5
**12**			Horizontal line sampling	1	5
**13**			Single corner sampling	5	1
**14**			Impartial sampling	1	5

### Pigsties

A typical sized farrow-to-finish type pig farm in Japan has almost 200 sows and 1800 suckling or fattening pigs [11]. Such pig farms have multiple pigsties, and in general, these pigsties can be divided into three types: (i) individual stalls for immature or pregnant sows, (ii) grouping pens for fattening pigs, and (iii) grouping pens for sows with suckling piglets. In this study, individual-stall and group-housing pigsties, which resemble pigsties for sows and fattening pigs, respectively, were examined. Considering the pigsties in a typical farrow-to-finish type farm in Japan (personal communication), the number of pigs and the layout and the size of stalls or pens in a pigsty were assumed as shown in Table 2.

**Table 2:** Conditions of the virtual pigsties.

Type of the pigsty	Number of pigs per pigsty	Number of pigs per stall or pen	Number of stalls or pens per pigsty	Number of stalls or pens per line	Number of lines per pigsty	Length of stall or pen (m)	Width of stall or pen (m)	Width of path (m)
**Stall-housing pigsty**	250	1	250	50	5	2.2	0.65	0.9
**Group-housing pigsty**	600	15	40	10	4	5.4	2.7	0.9

### Infection pattern in a pigsty

**Fig 1:**
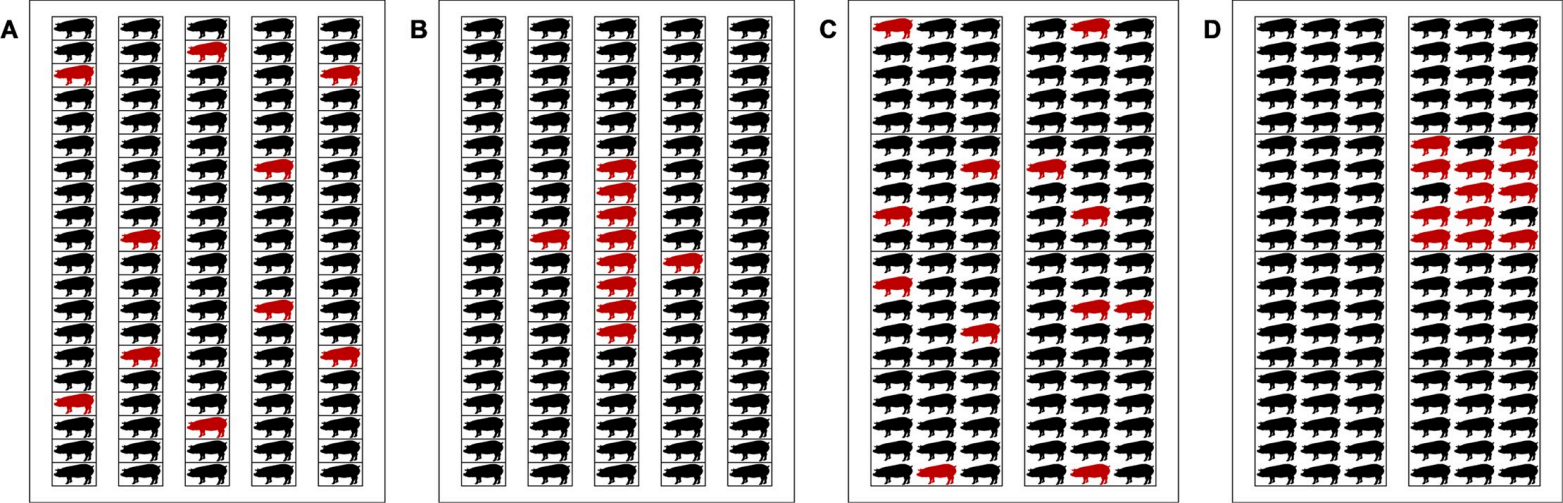
Illustration of the distribution of infected pigs in a pigsty. Pigs in red are infected and pigs in black are not infected. (A) The scattered infection scenario in the stall-housing pigsty. (B) The localized infection scenario in the stall-housing pigsty. (C) The scattered infection scenario in the group-housing pigsty. (D) The localized infection scenario in the group-housing pigsty.

To examine the influence of the idealistic assumption of a homogeneous distribution of infected pigs on the outputs, two infection scenarios were included in this study: scattered infection and localized infection. A localized infection resembles an infection in a real situation, whereas a scattered infection resembles a hypothetical infection. In the scattered infection scenario, infected pigs were assumed to be distributed randomly in a pigsty (Fig 1). In a localized infection scenario in an individual-stall housing pigsty, infected pigs were assumed to be distributed locally around the pig that was the source of infection, and was randomly located in a pigsty (Fig 1). In the localized infection scenario in a group-housing pigsty, the source of infection (pig) was randomly selected, and infection was assumed to spread to pigs within the pen (Fig 1). If the number of pigs inside was less than the desired number of infected pigs, additional infected pigs were selected from one of the adjacent pens. The number of infected pigs was calculated according to the assumed prevalence to fit the pigsty type. The disease prevalence was changed from the range of 5–95% with 5% intervals. Table 3 shows the number of infected pigs/pigsty calculated according to the given prevalence.

**Table 3:** Number of infected pigs according to disease prevalence.

Type of the pigsty	Disease prevalence (%)																		
	5	10	15	20	25	30	35	40	45	50	55	60	65	70	75	80	85	90	95
**Stall-housing pigsty**	13	25	38	50	63	75	88	100	113	125	138	150	163	175	188	200	213	225	238
**Group-housing pigsty**	30	60	90	120	150	180	210	240	270	300	330	360	390	420	450	480	510	540	570

### Sampling methods

Four sampling methods, namely, random sampling, two types of localized sampling, and impartial sampling, were examined in this study (Table 4). The two localized sampling methods involve the selection of adjacent pigs in horizontal or vertical directions; these methods are less laborious and do not involve random selection. This method was examined because it was actually used for testing some farms during the CSF outbreak in Japan. In the impartial sampling method, all the areas of a pigsty were covered without performing random selection.

**Table 4:** Descriptions of sampling methods.

Sampling method	Description
**Stall-housing pigsty**	
Random sampling	Selecting five pigs from the pigsty by simple random sampling
Localized sampling	Selecting five consecutive pigs from one corner of the pigsty along stall line (vertical line sampling) or across stall lines (horizontal line sampling)
Impartial sampling	Selecting five pigs from four corners and the center of the pigsty
**Group-housing pigsty (sampling one pig per pen)**	
Random sampling	Selecting five pens from the pigsty by simple random sampling and subsequently selecting one pig from each pen by simple random sampling
Localized sampling	Selecting five consecutive pens from one corner of the pigsty along (vertical line sampling) or across pen lines (horizontal line sampling) and subsequently selecting one pig from each pen by simple random sampling
Impartial sampling	Selecting five pens from four corners and the center of the pigsty and subsequently selecting one pig from each pen by simple random sampling
**Group-housing pigsty (sampling five pigs from one pen)**	
Random sampling	Selecting one pen from the pigsty by simple random sampling and subsequently selecting five pigs from the pen by simple random sampling
Localized sampling	Selecting one pen locating in one corner of the pigsty and subsequently selecting five pigs from the pen by simple random sampling (single corner sampling)

In the contingency plan for CSF in Japan, the sample size/pigsty for testing the farms associated with or surrounding the infected farm was prescribed to be at least five [4]. Thus, in this study, the number of pigs to be tested per pigsty was set at five.

### Detecting infection

Both, the sensitivity and the specificity of the test were assumed to be 100%. Although this assumption appears unrealistic, our study aimed to compare the relative sensitivity of the surveillance conducted using different sampling methods, and thus, this unrealistic assumption was not expected to affect its reliability. Detecting one or more infected pigs was judged as the successful detection of an infected farm, because detection of an infected pig always leads to the slaughter of all pigs in the detected farm.

### Monte Carlo simulation

We tested 10,000 iterations of infection and sampling for every combination of simulation scenario and disease prevalence to calculate the probability of detecting infected farms. Table 1 shows the 14 scenarios that were examined considering the structure of the pigsty, distribution of infected pigs, sampling method, and the number of pig(s) to be tested per pen in a group-housing pigsty. All the simulations were performed using R, version 3.5.1.

### Sensitivity analysis

Sensitivity analysis was conducted to examine the influence of uncertainty, that was not considered by the original scenarios and other assumptions applied in this study. Possible variations in the size of a pigsty, the layout of stalls or pens in a pigsty, the number of pigs to be tested per pigsty, and the size of pens (in the case of group-housing pigsty) were tested in the sensitivity analysis. The pattern of infection was fixed as localized infection, and four sampling methods were evaluated under each assumption: random sampling, vertical line sampling, horizontal line sampling, and impartial sampling. Settings and values included in the sensitivity analysis are listed in the S1 and S2 Tables.

## Results

### Sampling in stall-housing pigsties

**Fig 2:**
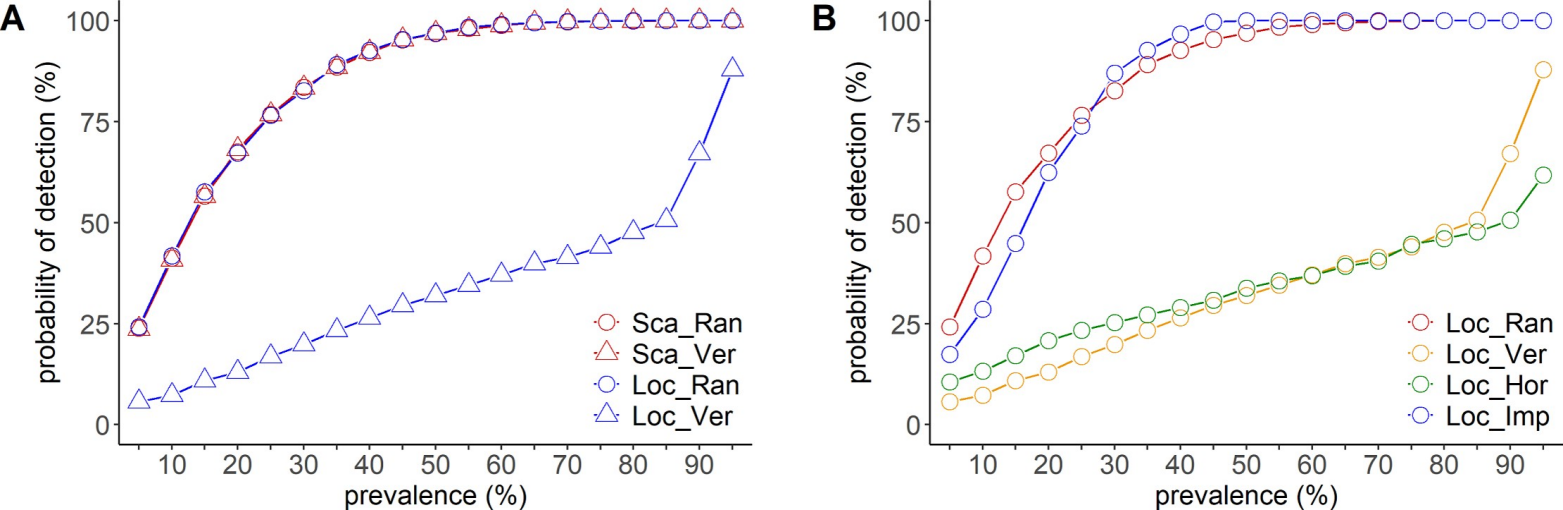
Relationship between disease prevalence and the probability of detection in a stall-housing pigsty. (A) Comparison of the probability of detection according to infection pattern. Scattered infection is indicated in red and localized infection is indicated in blue. The sampling method is represented by circles (random sampling) and triangles (vertical line sampling; as a representative of localized sampling). (B) Comparison of the probability of detection according to the sampling method under the localized infection scenario. Random sampling is indicated in red, vertical line sampling is indicated in yellow, horizontal line sampling is indicated in green, and impartial sampling is indicated in blue.

Fig 2 shows the changes in the probability of detecting infection depending on the sampling method used in a stall-housing pigsty. Under the scattered infection scenario, random sampling and localized sampling showed equal probability of detecting infection. The probability reached almost 95% when disease prevalence was set at 45%. Random sampling showed almost equal probability of detection, even in the localized infection scenario. However, in the localized infection scenario, the localized sampling method showed a lower probability of detection than the random sampling method, irrespective of the sampling direction. Impartial sampling and random sampling showed a comparable probability of detection in both, scattered and localized infection scenarios.

### Sampling in group-housing pigsties

**Fig 3:**
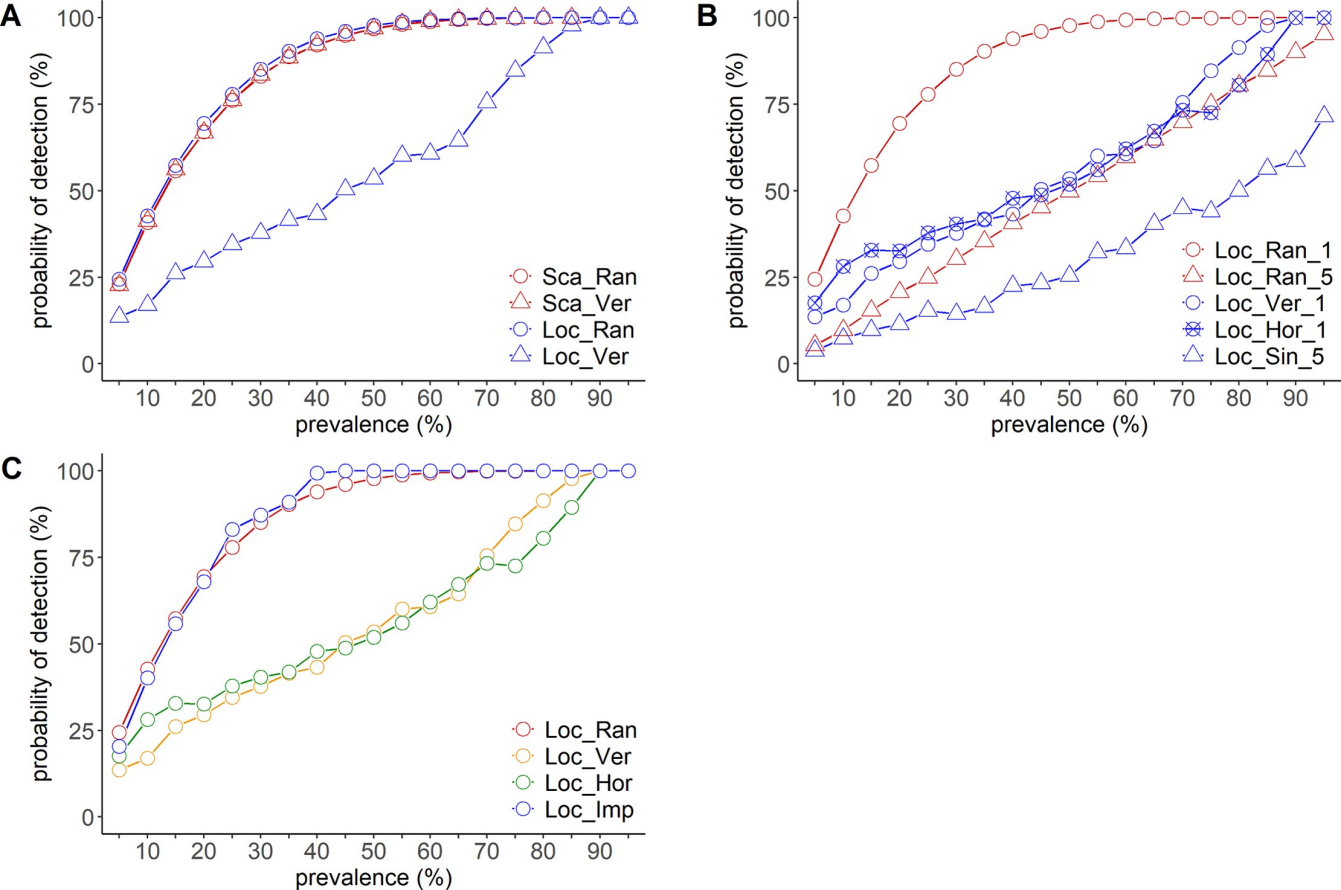
Relationship between disease prevalence and the probability of detection in a group-housing pigsty. (A) Comparison of the probability of detection according to infection pattern. Five pigs were selected from different pens. The scattered infection scenario is indicated in red and the localized infection scenario is indicated in blue. The sampling method is indicated by circles (random sampling) and triangles (vertical line sampling, as a representative example of localized sampling). (B) Comparison of the probability of detection according to the number of the tested pig(s) per pen under the localized infection scenario. Random sampling is indicated in red and localized sampling is indicated in blue. The number of tested pigs per pen is indicated by a circle (one pig per pen), a circle with a cross (one pig per pen), and a triangle (five pigs per pen). A circle in blue represents vertical line sampling, a circle with a cross in blue represents horizontal line sampling, and a triangle in blue shows single corner sampling. (C) Comparison of the probability of detection according to the sampling method under the localized infection scenario. Five pigs were selected from different pens. Random sampling is indicated in red, vertical line sampling is indicated in yellow, horizontal line sampling is indicated in green, and impartial sampling is indicated in blue.

Fig 3 shows the probability of detecting infection depending on the group-housing pigsty scenarios. Under the scattered infection scenario, random sampling and localized sampling showed equal probabilities of detection. The probability reached almost 95% with 45% disease prevalence. Random sampling showed equal probability of detection under both, scattered and localized infection scenarios. Sampling one pig/pen showed a higher probability of detection than sampling five pigs from one pen, irrespective of the sampling method used. Under the localized infection scenario, localized sampling showed a lower probability of detection than random sampling, irrespective of the sampling direction. Impartial sampling and random sampling showed comparable probabilities of detection.

### Sensitivity analysis

The results of sensitivity analysis are shown in the S1 and S2 Figs. In the stall-housing pigsty, impartial sampling and random sampling always showed higher probabilities of detection than localized sampling, irrespective of the pigsty size, layout of stalls, and sample size. In the group-housing pigsty, impartial sampling and random sampling always showed higher probabilities of detection than localized sampling, irrespective of the size of pigsty and pen, layout of pens, sample size, and number of pigs to be tested per pen. Although impartial sampling showed lower probabilities of detection than random sampling in some layouts of stalls or pens in both type of pigsties, the difference was small, and the probabilities of detection with both these sampling methods were always higher than those with localized sampling methods.

## Discussion

The probability of detecting infected farms by emergency surveillance conducted during an exotic infectious disease outbreak depends on how the animals to be sampled are selected. To provide evidence that will help veterinary officials conducting farm visits for sampling, probabilities of detection by different sampling methods were compared using a simulation method. The influence of localization of infected pigs in pigsties and differences in pigsty types, i.e. stall type and group-housing type, were also considered.

As an idealistic situation, sampling under scattered infection scenario was assumed in this study. Under the scattered infection scenario, random sampling and localized sampling showed equal probabilities of detection; this reached almost 95% with 45% disease prevalence, respectively, regardless of the type of pigsty. These results are similar to those obtained using five samples and assuming binomial probability [12], because this equation assumes randomness in both, infection and sample selection. Under the scattered infection scenario, the weakness of the localized sampling method appears to be cancelled because each sample has an equal chance to contain infected pigs, irrespective of the location of the sample. In contrast, under the localized infection scenario, the probability of detection differed between sampling methods, even at the same prevalence. When a disease such as CSF infects one pig in a pigsty, it initially spreads to the pigs that are adjacent to the initially infected pig, and results in the localized distribution of infected pigs in a pigsty. This implies that an assumption of scattered infection, applied for the emergency surveillance of exotic diseases, may not be optimal; selecting appropriate sampling measures is critical in a realistic situation.

Random sampling showed a relatively high probability of detection under both, scattered and localized infection scenarios and a higher probability of detection than the other sampling methods at almost any disease prevalence (especially in low prevalence), irrespective of the type of pigsty. When random sampling is conducted, all pigs should be listed with their location inside a pigsty and individually identified with specific identification numbers [13]. However, in Japan, piglets and fattening pigs are not usually identified, and even the location of sows, which often have been identified, are not recorded. Conducting proper random sampling in such pig farms is time-consuming, as veterinary officials have to start identifying all pigs with their locations. Alternatively, any localized sampling method can be applied for less laborious sampling.

Collecting samples from a localized part of a pigsty in a horizontal or vertical manner was studied as a labor-efficient sampling method, because pig identification is not necessary in these methods. In the localized infection scenario, these methods show lower probabilities of detection than random sampling and impartial sampling, irrespective of the type of pigsty. As the samples from nearby pigs tend to have the same test results in the localized infection scenario, test results from each sample are expected to be highly correlated and lack representativeness. This leads to a lower probability of detection with these sampling methods and defeats the purpose of emergency surveillance, i.e. early detection of infected farms.

As an alternative to the random sampling method, the impartial sampling method, i.e., the selection of pigs evenly from the pigsty, was proposed in this study. To simplify the operation of sampling, the impartial sampling method involves the collection of samples from the four corners and the center of the pigsty without pig identification and random selection. Under the localized infection scenario, the impartial and random sampling methods in both types of pigsties showed comparable probabilities of detection. In the impartial sampling method, all samples are evenly distributed inside a pigsty, and it resembles the systematic random sampling method, i.e., pigs at fixed intervals in the pigsty are selected. Therefore, impartial and random sampling may have comparable effectiveness. This method is less laborious with a high probability of detection, and may be a good alternative to the random sampling method in emergency surveillances.

For group-housing pigsties, in which usually piglets or fattening pigs are raised, two different sampling approaches may be considered when five samples per pigsty are sampled. One is collecting five samples from one pen and the other is collecting one sample from each of the five pens. Under the localized infection scenario, sampling one pig per pen showed a higher probability of detection than sampling all five pigs from one pen, irrespective of the sampling methods. Pigs always have direct contact with other pigs within the same pen; however, they have lower chances of being in contact with pigs from other pens, directly or indirectly. Previous transmission experiments on CSF found that the infection rate was higher for within-pen transmission than for between-pen transmission [14]. Therefore, as mentioned previously, localized sampling has a lower probability of detection; five samples in the same pen will have higher similarity and less representativeness. Thus, the probability of detection in five-samples-in-one-pen strategy is lower than that of one-sample-in-five-pens strategy. For sampling conducted in a group-housing pigsty, sampling one pig from each of five pens is strongly recommended for emergency surveillance.

Even if the recommended sampling methods are applied, the probability of detection is very low when the disease prevalence is low within the tested pigsty. Although the sensitivity analysis showed that the probability of detection was improved by increasing the number of samples collected for a pigsty, the available resources in an emergency will limit the sample size. This indicates the importance of repeated sampling before lifting movement restrictions against the farms subjected to emergency surveillance, which enables the detection of infection if the disease spreads within/between the pigsties to detectable levels. During the CSF outbreak in Japan, all targeted farms (except for several early cases) were repeatedly tested on days 0, 17, and 26 after the detection of the initial infected farm [3, 4].

The influence of assumptions that were not considered in the original scenario, such as the size of the pigsty and pen, layout of stalls (or pens), sample size, and the number of pigs to be tested per pen, was evaluated by sensitivity analysis. Even with these assumptions during sensitivity analysis, impartial sampling and random sampling always showed a higher probability of detection than localized sampling in both types of pigsties. Impartial sampling showed a lower probability of detection than random sampling in some layouts of stalls (or pens); however, the difference was small, and its superiority over localized sampling did not change.

Under the localized infection scenario, infection was assumed to spread only to the pigs adjacent to the source pig. In addition, in the group-housing pigsty, between-pen transmission was assumed to occur after all pigs in the source-of-infection pen were infected. However, actual infectious diseases can be transmitted to distant pigs and may spread to the other pens before all pigs in the source-of-infection pen are infected. In this study, we examined a more relaxed situation of localized infection, i.e., we randomly selected infected pigs from those that were close to, but not necessarily adjacent to or within the same pen as the source-of-infection pig; however, this provided the same results (see S1 Appendix). In general, detecting an infected animal will be much easier when an infection occurs in a more scattered manner. Therefore, the localized infection assumed in this study will bring the most conservative results for the probability of detecting infected farms.

The assumption of perfect test sensitivity and specificity for the test applied in the simulation is rather unrealistic. However, our study aimed to compare the probability of detection between the different sampling methods. Thus, this assumption did not interfere with our results when applied to all the scenarios examined in this study.

Under the realistic localized infection scenario, random sampling and impartial sampling showed comparable probabilities of detection, that were higher than those of other sampling methods. Additionally, impartial sampling is relatively labor-efficient, and thus, may be a substitute for random sampling. From a viewpoint of risk-based surveillance, animals showing clinical signs should be the first targets of sampling [15]. However, in the case of CSF that shows fewer clinical signs, as reported in the recent outbreak in Japan, such a risk-based approach will not be feasible. Moreover, during an outbreak of animal infectious diseases, farms surrounding or having epidemiological connections with infected farms are generally targeted for active surveillance, irrespective of the absence of any clinical signs in the pigs. In such conditions, use of sampling methods with higher probability of detection and labor efficiency is strongly recommended. The results of this study provide useful evidence for official veterinarians and institutes conducting emergency surveillance of infectious diseases for selecting preferable surveillance methods.

## Supporting information

S1 Fig Results of sensitivity analysis in the stall-housing pigsty. Heatmap from top to bottom representing the probability of detection by vertical line sampling, horizontal line sampling, impartial sampling, and random sampling are shown in each figure from (a) to (c). On the left side of the heatmaps, "H" stands for head (the number of pigs per line), "L" stands for line (the number of lines per pigsty), and "S" stands for sample size. (a) Comparison of the sampling methods according to the pigsty size (the number of pigs per pigsty: 90, 250, and 490). (b) Comparison of the sampling methods according to the stall layout (the number of pigs per pigsty was unified as almost 250). (c) Comparison of the sampling methods by sample size (samples size: 5, 7, and 9).
(JPEG)

S2 Fig Results of sensitivity analysis in the group-housing pigsty. Heatmap from top to bottom representing the probability of detection by vertical line sampling, horizontal line sampling, impartial sampling, and random sampling are shown in each figure from (a) to (d). on the left side of the heatmaps, "H" stands for head (the number of pigs per pen), "P" stands for pen (the number of pens per line), "L" stands for line (the number of lines per pigsty), and "S" stands for sample size. (a) Comparison of the sampling methods according to pen size (the number of pigs per pen: 10, 15, and 30). (b) Comparison of the sampling methods according to pigsty size (the number of pigs per pigsty: 150, 600, and 1350). (c) Comparison of the sampling methods according to pen layout (the number of pigs per pigsty was unified as 600). (d) Comparison of the sampling methods according to sample size (sample size: 5, 7, and 9).
(JPEG)

S1 Table Conditions of a stall-housing pigsty for sensitivity analysis. Underlined conditions are same as the original scenario.
(DOCX)

S2 Table Conditions of a group-housing pigsty for sensitivity analysis. Underlined conditions are same as the original scenario.
(DOCX)

S1 Appendix The results of simulation assuming “relaxed” localized infection; infection can occur in non-adjacent pens with low probability. (DOCX)
