# Supplementary figures and images for: Evaluation of sampling methods for effective detection of infected pig farms during a disease outbreak
Source: PLoS One. 2020 Oct 22;15(10):e0241177. doi: 10.1371/journal.pone.0241177 (PMC7580991; doi:10.1371/journal.pone.0241177)

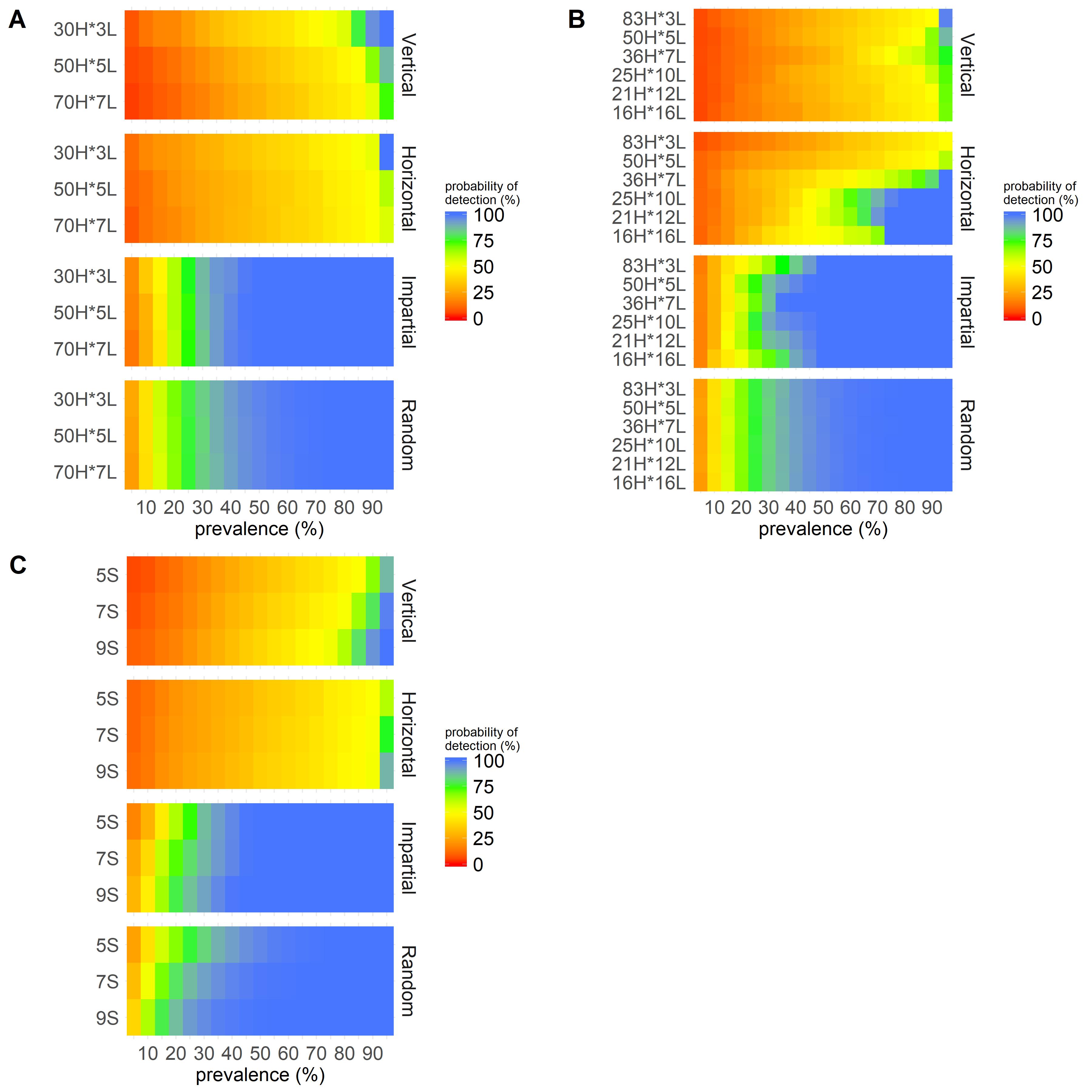

Supplement: S1 Fig — Heatmap from top to bottom representing the probability of detection by vertical line sampling, horizontal line sampling, impartial sampling, and random sampling are shown in each figure from (a) to (c). On the left side of the heatmaps, "H" stands for head (the number of pigs per line), "L" stands for line (the number of lines per pigsty), and "S" stands for sample size. (a) Comparison of the sampling methods according to the pigsty size (the number of pigs per pigsty: 90, 250, and 490). (b) Comparison of the sampling methods according to the stall layout (the number of pigs per pigsty was unified as almost 250). (c) Comparison of the sampling methods by sample size (samples size: 5, 7, and 9). (JPEG) [file pone.0241177.s001.jpeg]

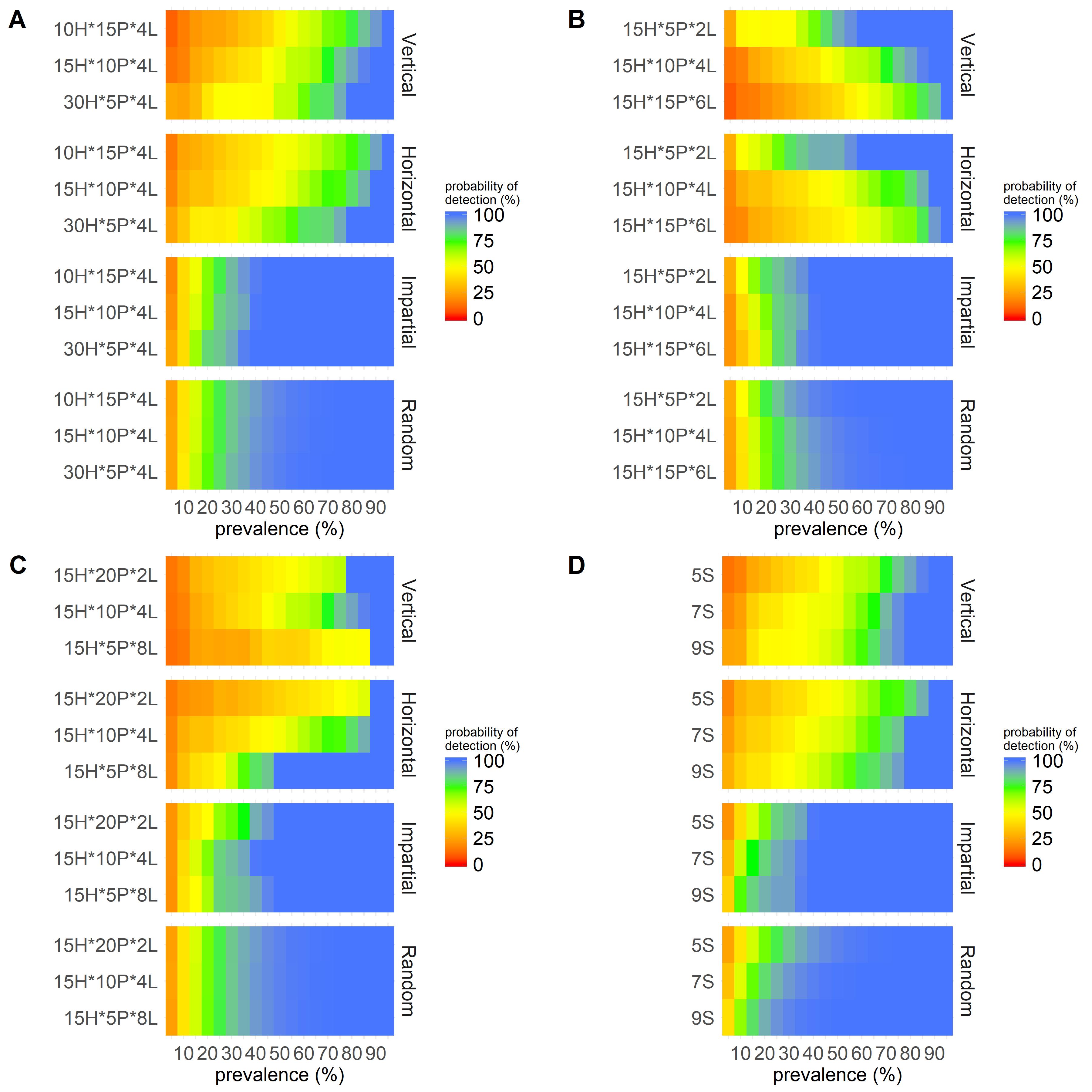

Supplement: S2 Fig — Heatmap from top to bottom representing the probability of detection by vertical line sampling, horizontal line sampling, impartial sampling, and random sampling are shown in each figure from (a) to (d). on the left side of the heatmaps, "H" stands for head (the number of pigs per pen), "P" stands for pen (the number of pens per line), "L" stands for line (the number of lines per pigsty), and "S" stands for sample size. (a) Comparison of the sampling methods according to pen size (the number of pigs per pen: 10, 15, and 30). (b) Comparison of the sampling methods according to pigsty size (the number of pigs per pigsty: 150, 600, and 1350). (c) Comparison of the sampling methods according to pen layout (the number of pigs per pigsty was unified as 600). (d) Comparison of the sampling methods according to sample size (sample size: 5, 7, and 9). (JPEG) [file pone.0241177.s002.jpeg]
