# Supplementary material for: Evaluation of sampling methods for effective detection of infected pig farms during a disease outbreak
Source: PLoS One. 2020 Oct 22;15(10):e0241177. doi: 10.1371/journal.pone.0241177 (PMC7580991; doi:10.1371/journal.pone.0241177)
Supplement: S1 Table — Underlined conditions are same as the original scenario. (DOCX) [file pone.0241177.s003.docx]

**S1 Table. Conditions of a stall-housing pigsty for sensitivity analysis.** Underlined conditions are same as the original scenario.

| **Viewpoint** | **Number of pigs per pigsty** | **Number of pigs per stall** | **Number of stalls per pigsty** | **Number of stalls per line** | **Number of lines per pigsty** | **Length of stall (m)** | **Width of stall (m)** | **Width of path (m)** | **Number of tested pigs per stall** | **Number of tested stalls** |
| --- | --- | --- | --- | --- | --- | --- | --- | --- | --- | --- |
| Pigsty size | 90 | 1 | 90 | 30 | 3 | 2.2 | 0.65 | 0.9 | 1 | 5 |
|  | 250 | 1 | 250 | 50 | 5 | 2.2 | 0.65 | 0.9 | 1 | 5 |
|  | 490 | 1 | 490 | 70 | 7 | 2.2 | 0.65 | 0.9 | 1 | 5 |
| Stall layout | 249 | 1 | 249 | 83 | 3 | 2.2 | 0.65 | 0.9 | 1 | 5 |
|  | 250 | 1 | 250 | 50 | 5 | 2.2 | 0.65 | 0.9 | 1 | 5 |
|  | 252 | 1 | 252 | 36 | 7 | 2.2 | 0.65 | 0.9 | 1 | 5 |
|  | 250 | 1 | 250 | 25 | 10 | 2.2 | 0.65 | 0.9 | 1 | 5 |
|  | 252 | 1 | 252 | 21 | 12 | 2.2 | 0.65 | 0.9 | 1 | 5 |
|  | 256 | 1 | 256 | 16 | 16 | 2.2 | 0.65 | 0.9 | 1 | 5 |
| Sample size | 250 | 1 | 250 | 50 | 5 | 2.2 | 0.65 | 0.9 | 1 | 5 |
|  | 250 | 1 | 250 | 50 | 5 | 2.2 | 0.65 | 0.9 | 1 | 7 |
|  | 250 | 1 | 250 | 50 | 5 | 2.2 | 0.65 | 0.9 | 1 | 9 |
