# Supplementary material for: Evaluation of sampling methods for effective detection of infected pig farms during a disease outbreak
Source: PLoS One. 2020 Oct 22;15(10):e0241177. doi: 10.1371/journal.pone.0241177 (PMC7580991; doi:10.1371/journal.pone.0241177)
Supplement: S2 Table — Underlined conditions are same as the original scenario. (DOCX) [file pone.0241177.s004.docx]

**S2 Table. Conditions of a group-housing pigsty for sensitivity analysis.** Underlined conditions are same as the original scenario.

| **Viewpoint** | **Number of pigs per pigsty** | **Number of pigs per pen** | **Number of pens per pigsty** | **Number of pens per line** | **Number of lines per pigsty** | **Length of pen (m)** | **Width of pen (m)** | **Width of path (m)** | **Number of tested pigs per pen** | **Number of tested pens** |
| --- | --- | --- | --- | --- | --- | --- | --- | --- | --- | --- |
| Pen size | 600 | 10 | 60 | 15 | 4 | 5.4 | 1.8 | 0.9 | 1 | 5 |
|  | 600 | 15 | 40 | 10 | 4 | 5.4 | 2.7 | 0.9 | 1 | 5 |
|  | 600 | 30 | 20 | 5 | 4 | 5.4 | 5.4 | 0.9 | 1 | 5 |
| Pigsty size | 150 | 15 | 10 | 5 | 2 | 5.4 | 2.7 | 0.9 | 1 | 5 |
|  | 600 | 15 | 40 | 10 | 4 | 5.4 | 2.7 | 0.9 | 1 | 5 |
|  | 1350 | 15 | 90 | 15 | 6 | 5.4 | 2.7 | 0.9 | 1 | 5 |
| Pen layout | 600 | 15 | 40 | 20 | 2 | 5.4 | 2.7 | 0.9 | 1 | 5 |
|  | 600 | 15 | 40 | 10 | 4 | 5.4 | 2.7 | 0.9 | 1 | 5 |
|  | 600 | 15 | 40 | 5 | 8 | 5.4 | 2.7 | 0.9 | 1 | 5 |
| Sample size | 600 | 15 | 40 | 10 | 4 | 5.4 | 2.7 | 0.9 | 1 | 5 |
|  | 600 | 15 | 40 | 10 | 4 | 5.4 | 2.7 | 0.9 | 1 | 7 |
|  | 600 | 15 | 40 | 10 | 4 | 5.4 | 2.7 | 0.9 | 1 | 9 |
