# Supplementary material for: Evaluation of sampling methods for effective detection of infected pig farms during a disease outbreak
Source: PLoS One. 2020 Oct 22;15(10):e0241177. doi: 10.1371/journal.pone.0241177 (PMC7580991; doi:10.1371/journal.pone.0241177)
Supplement: S1 Appendix — (DOCX) [file pone.0241177.s005.docx]

**The results of simulation** **assuming “relaxed” localized infection; infection can occur in non-adjacent pens with low probability**

Under the “relaxed” localized infection scenario, we assumed that infection can occur even in the pens not located next to the infected pen, with a higher probability of infection in the closer pens.

The figures below show the changes in the probability of detecting infection depending on the sampling method used in a stall-housing pigsty (Fig S1A-1) and a group-housing pigsty (Fig S1A-2).

**Fig S1A-1. Relationship between disease prevalence and the probability of detection in a stall-housing pigsty.** Comparison of the probability of detection according to the sampling method under the “relaxed” localized infection scenario. Random sampling is indiacted in red, vertical line sampling is indicated in yellow, horizontal line sampling is indicated in green, and impartial sampling is indicated in blue.

**Fig S1A-2. Relationship between disease prevalence and the probability of detection in a group-housing pigsty.** (A) Comparison of the probability of detection according to the sampling method under the “relaxed” localized infection scenario. Five pigs were selected from different pens. Random sampling is indicated in red, vertical line sampling is indicated in yellow, horizontal line sampling is indicated in green, and impartial sampling is indicated in blue. (B) Comparison of the probability of detection according to the number of the tested pig(s) per pen under the “relaxed” localized infection scenario. Random sampling is indicated in red and localized sampling is indicated in blue. The number of tested pigs per pen is represented by a circle (one pig per pen), a circle with a cross (one pig per pen), and a triangle (five pigs per pen). A circle in blue represents vertical line sampling, a circle with a cross in blue represents horizontal line sampling, and a triangle in blue shows single corner sampling.
